# On the Origin of the Cow-Pox, with a Few Remarks on the Pox of Swine

**Published:** 1799-04

**Authors:** J. Lawrence


FOR THE MEDICAL AND PHYSICAL JOURNAL.

### *On the Origin of the Cow-pox, with a few Remarks on the Pox of Swine.*

(By Mr. Lawrence, Author of a 'Philoſophical and Practical Treatiſe on Horſes, and on the Moral Duties of Man towards the Brute Creation,' 2 vol. 8vo.—&c. &c.)

IT is in conſequence of particular requeſts, that I reſume my pen, to offer a few practical remarks on the origin of a diſeaſe, which a recent, and moſt ingenious diſcovery, has rendered ſo generally intereſting. I have not the preſumption to ſuppoſe, that my obſervations, although by no means lightly made, can be of any conſiderable moment; but in a matter of public concern, a man's endeavours evince, at leaſt, his alacrity and good-will; and I am proud to contribute my mite, amongſt the more important, and ſtrongly-grounded opinions, of ſcientific and profeſſional men, ſo much more amply prepared for the inveſtigation.

In the firſt place, I muſt beg leave to differ conſiderably in opinion, from a reſpectable correſpondent of the Journal, Dr. Sims, who intimates that "all hazardous experiments ought to be diſcouraged." Such a poſition, I think, is too much in the ſpirit of ancient prejudice, the grand retarder of all improvement, which can ſeldom make a progreſs without conſiderable riſk, and preſent inconvenience. Upon ſuch principles, inoculation for the ſmall-pox, which has preſerved ſo many valuable lives, and contributed ſo much to the health and perſonal appearance of ſuch numbers, in this country particularly, cularly, would have been, at this day, unknown and unpractifed. Every one, who has read upon this fubject, well knows the ftrenuous oppofition which the firft inoculators had to encounter, both in England and America, from vulgar prejudice; and that fome of them, with their patients, found a difficulty even to efcape with their lives. Dr. Sims has adduced a fingle inftance of inveterate and loathfome cow-pox, and of its failure as a preventive of the fmall-pox. Without doubt, all due attention ought, and will be paid to this inftance, but no more. Examples on the favourable fide are much more numerous and precife. There have been alfo reports, apparently well-founded, of a recurrence of the fmall-pox. As to the peculiar loathfomenefs of the cow-pox, all infectious and eruptive difeafes are fufficiently fo; but with refpect to the natural purity of the animal juices, none can be more truly fo, or more wholefome, than thofe of the cow. In this affair, the medical faculty have ftrictly performed their duty, both as good patriots, and as men eager for the propagation of fcience. The difcovery is fully and fairly in the way of experiment, and will ftand or fall by its own merits.

My remarks on the prefumed origin of the difeafe, namely, that it proceeds from the greafe of horfes' heels, inoculated into the teats of cows, I hope, will not be conftrued into any difrefpect, or affected oppofition to Dr. Jenner, whofe abilities and activity in profeffional purfuits, I admire, and to whofe phyfical opinions I feel difpofed to pay the utmoft deference. Indeed, I am inclined to fuppofe the Doctor publifhed that fingular hypothefis, rather out of compliance with the ideas of the people he was obliged to confult, than as the refult of his own mature reflection. A very flight confideration would determine, *a priori*, the utter improbability of the fact, had not its impoffibility been already proved, by a fufficient number of accurate experiments*. It is in oppofition to all analogy, and to the invariable determinations of nature, to fuppofe, that a fimple, and innoxious humour, fhould poffefs the power of generating infection. Such a humour is the greafe, primarily, in the heels of horfes. The eruption of this humour is generally occafioned by defective abforption and circulation in the lower extremities of the animal, whilft continued long within doors, in a ftanding pofture, and confequent ftagnation: its prevention—exercife, and much friction of the legs by the groom, with a minute attention to cleanlinefs, or voluntary exercife abroad. This is the cafe with all horfes, in a greater or lefs degree: and examples of thofe in a found and healthy ftate, greafed *in a few days*, by ignorant and incapable grooms, are innumerable.

Dr. Jenner

* See Mr. Simmons, on cow-pox, &c.

Dr. Jenner, however, no doubt aware of this impediment, obferves—"Horfes with heels difeafed in that peculiar way, which I conceive to produce cow-pox, were fo rarely met with, during the progrefs of the experiments as not to be within my reach." The Doctor rationally expected a degree of acrimony fufficient for the purpofe of infection, in the matter of the chronic and inveterate greafe alone. There is no novelty or doubt in regard to the virulence of corrupt and ftagnant fluids; nor is it at all improbable, but an animal poifon, communicated or inoculated, fhould infect animal juices; but that the fowing thefe feeds fhould produce a difeafe, in nature, fymptoms, and effects, totally diffimilar to the parent ftock, is at variance with all analogies, and therefore exceeds all belief.

Concerning the real ætiology of the difeafe, I have many years been without any uncertainty in my own opinion. The pox among cows I always fuppofed to arife from the contagion of their own atmofphere; a caufe fully adequate to the effect produced; and this effect ceafes with the caufe, or from the influence of fome cafual and unknown caufe, and is reproduced indefinitely with the recurrence of the original caufe. Too fudden repletion and thickening of the fluids, after the fatigue of long driving, and inanition, is an auxiliary and accelerating caufe of pox upon the teats, accompanied by low fever, confiderable debility, and a diminution both of the quantity and quality of the milk. This hypothefis, I think, will, at leaft, not be condemned as irrational, when it is confidered, that the difeafe is nearly, or altogether unknown, except in the dairy counties, and in large towns, where the animals, which are its victims, are congregated in fuch great numbers, and ftowed fo clofe.

The pox of fwine, called alfo by the London feeders, the *heavings*, appears to arife from the above-cited caufes precifely, but is attended with much feverer effects. I hope I may with truth affert, that I have loft more hogs and pigs by this difeafe, than any other man in Britain; by which I would be underftood, that I fhould be forry to hear of another who had been fo unlucky in that refpect. The eruption of puftules often covers the whole body; the fymptomatic fever prevails to a very exalted degree, the animal pants violently, and acquires a fhrill, hollow cry. Thofe are fatal fymptoms; but I have obferved fome to recover, which retained the ability of coughing ftrongly; a mean, perhaps, of difengaging, or rather preventing the lungs from adhering to the *pleura*. Such adhefion was generally obferved in thofe which died of the difeafe. In the milder ftages, the proper remedies are, feparation, air abroad, dry lodging, brimftone and madder, equal quantities twice a day, train-oil externally, warm food. I made repeated trials of of emetic tartar, and of crude mercury, in feveral bad pig cafes, without any perceptible effects.

To view the cow-pox inoculation in the flattering light, in which a rather extenfive range of experiment has already placed it, is to prognofticate much benefit to the human race from its benign operations. But thofe who are aware of the numerous former difappointments, from hypothetical fyftems of phyfics, and prefumed medical fpecifics, the infallability of thefe laft too, fo incontrovertibly proved by a great variety of experimental cafes, will not be unreafonably fanguine. They will be for a filent and fober appeal, from the fuccefs of early experiment, perhaps temporary, and a mere tranfitory deviation of nature from her general rule, to her more known and permanent courfe. By the inoculation of cow-pox, that difeafe alone is produced : is it not probable, that the feeds of variolous infection are only fmothered, or their activity fufpended, by the faturation of the juices with the vaccine virus ; and that, after a certain time, and the ceffation of fuch caufe of their fufpenfion, fome other may arife, proper to excite them into activity ?

The fingle cafe of Dr. Sims is a very ftrong one ; it is inconceivable that the patient, in that cafe, having the difeafe in fo violent a manner, fhould not have had it perfect, and with the fymptomatic fever, the prefumed condition on which no fmall-pox is afterwards to take place. Some of the accounts of Dr. Pearfon make the cow-pox a much more fevere difeafe than the inoculated fmall-pox, and take no notice of the fymptomatic fever. If thofe are to be depended upon, the cow-pox has already had its day. That it is often taken in fo flight a way, as to have no preventive effect of the fmall-pox, I apprehend pretty numerous proofs might be obtained.

But, granting the ultimate failure of this new fcheme, the public will have no great caufe of regret, fince the eftablifhed practice of inoculation is fo fuccefsful, and in fo happy a train of even farther improvement, from the meritorious labours of learned and experienced phyfiologifts, both of the continent, and of our own country. That fatal fcourge of the human race might, in all probability, by judicious and catholic meafures, be totally extirpated from civilized countries, in no very long period. Even could every family be induced by perfuafion, to inoculate all their children, whilft in arms, the difeafe would either finally ceafe, or become fo mild, as to be unattended with greater rifk than a mere common eruption.

Whatever may be the fate of cow-pox inoculation, it has, and will give further occafion to a pretty large and open difcuffion ; which is always beneficial, as having a tendency to produce difcovery, and promote improvement ; and when the public ardour for the prefent topic fhall have become a little cool.

cool and satisfied, I hope it will be turned, by enlightened men, towards another, perhaps of nearly as great consequence, namely, the prevention of the original malady in the animals themselves. Those who have witnessed, or only reflected upon, the excessive filth and nastiness, which must unavoidably mix with the milk, in an infected dairy of cows, and the corrupt and insalubrious state of their produce in consequence, will surely join me in that sentiment.

*March* 14, 1799. J. LAWRENCE.

